# Four-way decomposition of effect of cigarette smoking and body mass index on serum lipid profiles

**DOI:** 10.1371/journal.pone.0270486

**Published:** 2022-08-18

**Authors:** Wenhao Yu, Chaonan Gao, Xiangjuan Zhao, Chunxia Li, Bingbing Fan, Jiali Lv, Mengke Wei, Li He, Chang Su, Tao Zhang

**Affiliations:** 1 Department of Biostatistics, School of Public Health, Cheeloo College of Medicine, Shandong University, Jinan, Shandong, China; 2 Institute for Medical Dataology, Shandong University, Jinan, China; 3 National Institute of Health Data Science of China, Jinan, China; 4 Maternal and Child Health Care of Shandong Province, Cheeloo College of Medicine, Shandong University, Jinan, China; 5 National Institute for Nutrition and Health, Chinese Center for Disease Control and Prevention, Beijing, China; Tanta University Faculty of Medicine, EGYPT

## Abstract

**Objective:**

Smoking and obesity are established risk factors of dyslipidemia, however, the interplay between them has not been well studied. This study aims to explore the joint effect of smoking and body mass index (BMI) on serum lipid profiles.

**Methods:**

The study consisted of 9846 Chinese adults (mean age = 49.9 years, 47.6% males, 31.2% ever smokers), based on the China Health and Nutrition Survey. Serum lipid profiles included total cholesterol (TC), triglyceride (TG), low-density lipoprotein cholesterol (LDL-C), high-density lipoprotein cholesterol (HDL-C), apolipoprotein A (APO-A), and apolipoprotein B (Apo-B). The joint effect of smoking and BMI on serum lipids were examined by the four-way decomposition analysis and multivariate linear regression models.

**Results:**

The four-way decomposition showed that the interplay between smoking and BMI was complicated. There was only indirect effect (the mediated effect) between smoking and BMI on TC, LDL-C and APO-B. The pure indirect effect was -0.023 for TC, -0.018 for LDL-C, and -0.009 for APO-B. For TG, HDL-C and APO-A, the interaction effect was dominant. The reference interaction (the interactive effect when the mediator is left to what it would be in the absence of exposure) was 0.474 (P < 0.001) for TG, -0.245 (P = 0.002) for HDL-C, and -0.222 (P < 0.001) for APO-A, respectively. The effect of BMI on TG, HDL-C and APO-A were significantly higher in smokers than in nonsmokers (TG: 0.151 in smokers versus 0.097 in nonsmokers, HDL-C: -0.037 versus -0.027, APO-A: -0.019 versus -0.009, P for difference < 0.001 for all).

**Conclusion:**

These findings illustrate the joint effects of smoking and BMI on serum lipid profiles. There were significant interaction effects of smoking and BMI on TG, HDL-C and APO-A, while BMI maybe a mediator for the association of smoking with TC, LDL-C and APO-B. The effects between them were rather complex. Smoking cessation is necessary, especially for those overweight.

## Introduction

The prevalence of dyslipidemia was 33.8% overall among Chinese adults as reported in 2021 [1]. Dyslipidemia is a manageable risk factor for cardiovascular diseases (CVD), and serum lipids have been used to construct CVD prediction models [2, 3]. Therefore, the prevention and control of dyslipidemia is important to help decrease the occurrence of CVD.

Cigarette smoking is associated with plenty of chronic diseases. Previous studies have pointed that serum lipids (e.g., serum cholesterol, low-density lipoprotein) were higher in smokers than in non-smokers, except for high-density lipoprotein cholesterol (HDL-C) [4–6]. Abnormal blood lipids will further increase the risk of cardiovascular events, which may partly account for the strong correlation between smoking and cardiovascular diseases [7]. Obesity, as a well-known risk factor of CVD, is also correlated with dyslipidemia [8]. It has been proposed that obesity could result in higher fasting plasma triglycerides, low-density lipoprotein cholesterol (LDL-C) and lower HDL-C [9]. Recent researches suggested that obesity-induced dyslipidemia was not a unique pathophysiological entity, but rather distinct characteristics depending on many individual factors [10].

Although smoking and obesity are independently associated with chronic diseases, the interplay between them has not been well studied. It is well recognized that the relationship between smoking and BMI is complicated. Some studies have reported the interaction effect of smoking and body mass index (BMI) on all-cause and dyslipidemia mortality [11, 12]. California Teachers Study concluded that secondhand smoke was associated with increased risk of type II diabetes among non-smokers with obesity being a potentially important mediator [13]. Additionally, evidence showed that the decrease of dyslipidemia mortality in overweight/obese patients was due to the adverse causality and confounding bias associated with smoking [14]. Hence, it is necessary to assess mediation and interaction simultaneously. The four-way decomposition, a counterfactual approach, will help us to identify whether smoking could affect lipid profiles through obesity, or whether there is an interaction between smoking and BMI on lipid profiles [15].

Using data from the China Health and Nutrition Survey, the current study aims to explore the joint effect of cigarette smoking and BMI on serum lipid profiles by the four-way decomposition analysis.

## Method

### Study design

The China Health and Nutrition Survey (CHNS) is an ongoing longitudinal cohort implemented by national and local governments [16]. It is designed to understand how the social and economic transformation of Chinese society affects the health and nutritional status of Chinese population. A multi-stage, random cluster process was used to collect data from Beijing, Chongqing, Guangxi, Guizhou, Heilongjiang, Henan, Hubei, Hunan, Jiangsu, Liaoning, Shaanxi, Shandong, Shanghai, Yunnan, and Zhejiang. Nine cross-sectional surveys have been completed during 1989~2015, covering 4,400 households with 33,348 individuals.

The current study used the information of serum lipids (n = 10076) collected in CHNS 2009. Adult participants with stroke or myocardial infarction (n = 227) and missing values in smoking status (n = 3) were excluded. A total of 9846 participants (4685 males and 5161 females; mean age = 49.9 years) were included for the interaction analysis.

Study protocols were approved by the Institutional Review Committees of the University of North Carolina at Chapel Hill, NC, USA, and the China National Institute of Nutrition and Food Safety at the Chinese Center for Disease Control and Prevention, Beijing, China. Written informed consent was obtained from each study participants.

### Measurements

Standardized protocols were used by trained examiners. Standing height was measured without shoes to the nearest 0.2 cm using a portable SECA stadiometer (SECA, Hamburg, Germany). Weight in light clothing without shoes was measured to the nearest 0.1 kg on a dedicated scale that was routinely calibrated. BMI was calculated as weight in kilograms divided by height in meters squared.

All adult participants were required to collect 12ml blood (in three 4ml tubes) after overnight fasting. Total cholesterol (TC) was measured using CHOD-PAP, Kyowa (Japan). Triglycerides (TG) was measured using GPO-PAP, Kyowa (Japan). LDL-C and HDL-C were measured using Enzymatic method, Kyowa (Japan). Serum apolipoprotein A (APO-A) and apolipoprotein B (APO-B) were measured using Immunoturbidimetric method, Randox (UK).

Smoking was defined as ever smoking cigarettes. The mean daily energy intake(kcal) was obtained from the dietary intake data collected in the CHNS, which were derived from the Chinese food composition table. Educational attainment was classified as 7 categories: lower than/graduated from primary school, lower/upper middle school degree, technical or vocational degree, university or college degree, master’s degree or higher. Geographical regions were divided into rural and urban. Information on medication history and behavioral lifestyles were obtained in questionnaire survey.

The study protocols were approved by the Institutional Review Committees of the University of North Carolina at Chapel Hill, NC, USA, and the China National Institute of Nutrition and Food Safety at the Chinese Center for Disease Control and Prevention, Beijing, China (NO: 201524). Written informed consent was obtained from each study participants.

### Statistical analyses

Analyses of covariance were performed using generalized linear models to test differences in continuous study variables between smoking groups. TG was log-transformed for normal distribution. A four-way decomposition analysis [15] was used to examine whether there is a mediation or interaction effect between smoking and BMI on lipids. The total effect is decomposed into four components: (1) CDE (controlled direct effect), the direct effect of the exposure in the absence of the mediator; (2) INT_ref_ (the reference interaction), the interactive effect when the mediator is left to what it would be in the absence of exposure; (3) INT_med_, the mediated interaction, which will be significant when there is an interaction between mediator and exposure, and the exposure could affect the mediator; (4) PIE, the pure indirect effect, as well as the pure mediated effect. Note that this approach focuses more on the effect size rather than p value, to give recommendations for illustration. The effect of BMI on lipids was examined in smoking and non-smoking groups by multivariate linear regression models (R function: *lm*), separately. Statistical analyses were implemented with R version 3.6.3 and SAS version 9.4 (SAS Institute, Cary, NC).

## Results

**Table 1** summarizes the characteristics of the 9846 participants by smoking status. The mean levels of study variables were compared between smoking groups, adjusting for age (except age itself). The mean age of participants was 49.9 years. Among 3072 smokers, 93.6% were men. Smokers had lower BMI, LDL-C, HDL-C, and higher TG, energy intake than non-smokers. The proportion of educational levels were also different between two groups.

**Table 1:** Characteristics of participants by smoking status.

Variable	Nonsmoker	Smoker	Total	*P*-value
	(n = 6774)	(n = 3072)	(n = 9846)	
Age	49.6 (15.9)	50.5 (14.6)	49.9 (15.5)	0.011
Male, n (%)	1,809 (26.7)	2,876 (93.6)	4,685 (47.6)	< 0.001
BMI (kg/m^2^)	23.4 (3.54)	23.1 (3.31)	23.3 (3.47)	< 0.001
TC (mmol/L)	4.87 (1.02)	4.84 (0.97)	4.86 (1.01)	0.051
TG (mmol/L)	1.61 (1.40)	1.81 (1.66)	1.67 (1.49)	< 0.001
LDL-C (mmol/L)	2.99 (0.99)	2.93 (0.97)	2.97 (0.98)	< 0.001
HDL-C (mmol/L)	1.46 (0.48)	1.40 (0.53)	1.44 (0.50)	< 0.001
APO-A (g/L)	1.16 (0.35)	1.15 (0.47)	1.16 (0.39)	0.064
APO-B (g/L)	0.91 (0.27)	0.91 (0.26)	0.91 (0.27)	0.776
Energy (kcal)	2,051 (661.9)	2,302 (681.4)	2,130 (678.1)	< 0.001
Education, n (%)				
Lower than primary school	1,721 (25.4)	500 (16.3)	2,221 (22.6)	< 0.001
Primary school degree	1237 (18.3)	618 (20.2)	1,855 (18.9)	
Lower middle school degree	2117 (31.3)	1178 (38.4)	3,295 (33.5)	
Upper middle school degree	778 (11.5)	418 (13.6)	1,196 (12.2)	
Technical or vocational degree	501 (7.41)	219 (7.14)	720 (7.32)	
University or college degree	404 (5.97)	133 (4.34)	537 (5.46)	
Master’s degree or higher	6 (0.09)	1 (0.03)	7 (0.07)	
Region, n (%)				
Urban	2,320 (34.3)	1,022 (33.3)	3,342 (33.9)	0.307
Rural	4454 (65.8)	2050 (66.7)	6,504 (66.1)	

BMI = body mass index; TC = total cholesterol; TG = triglyceride; LDL-C = low density lipoprotein cholesterol; HDL-C = high density lipoprotein cholesterol; Apo-A = apolipoprotein A; Apo-B = apolipoprotein B

Continuous variables are presented as means (SD); *P*-values were adjusted for age.

**Table 2** presents the four-way decomposition of the effect of smoking on lipid profiles due to mediation and interaction with BMI, with adjustment for age, sex, region, energy intake, and education. The total effects of smoking on lipid profiles were 0.025 (*P* = 0.417) for TC, 0.046 (*P* = 0.019) for TG, 0.003 (*P* = 0.922) for LDL-C, -0.004 (*P* = 0.797) for HDL-C, 0.024 (*P* = 0.038) for APO-A and 0.012 (*P* = 0.124) for APO-B, respectively. The pure indirect effects were all statistically significant, but the reference interaction among four components was the most substantial. There was only indirect effect between smoking and BMI on TC, LDL-C and APO-B (-0.023, -0.018, and -0.009, respectively). For TG, HDL-C and APO-A, the interaction effect was dominant. INT_ref_, accounted for the interaction effect, was 0.474 (*P* < 0.001) for TG, -0.245 (*P* = 0.002) for HDL-C, and -0.222 (*P* < 0.001) for APO-A. INT_med_, the mediated interaction, was -0.010 (*P* = 0.001) for TG, 0.005 (*P* = 0.002) for HDL-C, and 0.005 (*P* = 0.002) for APO-A. Though the INT_med_ was also significant statistically, but the effect size was quite small contrast with the INT_ref_. We further performed a sensitivity analysis on the “current smoking”, the decomposition of the effect of current smoking on lipid profiles due to mediation and interaction with BMI was showed in **S1 Table**, though there were many missing values (6775 in 9846) in the variable “current smoking”, the results were basically consistent.

**Table 2:** Decomposition of the effect of smoking on lipid profiles due to mediation and interaction with BMI.

	TE		CDE		INT_ref_		INT_med_		PIE	
	Est (SE)	*P*-value	Est (SE)	*P*-value	Est (SE)	*P*-value	Est (SE)	*P*-value	Est (SE)	*P*-value
TC	0.025 (0.030)	0.417	-0.256 (0.163)	0.117	0.310 (0.162)	0.055	-0.006 (0.004)	0.077	-0.023 (0.005)	<0.001
TG	0.046 (0.020)	0.019	-0.390 (0.100)	<0.001	0.474 (0.099)	<0.001	-0.010 (0.003)	0.001	-0.027 (0.006)	<0.001
LDL-C	0.003 (0.029)	0.922	-0.003 (0.160)	0.986	0.025 (0.159)	0.877	-0.001 (0.003)	0.877	-0.018 (0.004)	<0.001
HDL-C	-0.004 (0.015)	0.797	0.222 (0.080)	0.005	-0.245 (0.079)	0.002	0.005 (0.002)	0.011	0.013 (0.003)	<0.001
APO-A	0.024 (0.011)	0.038	0.236 (0.062)	<0.001	-0.222 (0.062)	<0.001	0.005 (0.002)	0.005	0.004 (0.001)	<0.001
APO-B	0.012 (0.008)	0.124	-0.036 (0.043)	0.402	0.058 (0.042)	0.166	-0.001 (0.001)	0.185	-0.009 (0.002)	<0.001

TC = total cholesterol; TG = triglyceride; LDL-C = low density lipoprotein cholesterol; HDL-C = high density lipoprotein cholesterol; Apo-A = apolipoprotein A; Apo-B = apolipoprotein B

Est: the effect size of total effect or the component due to CDE, INTref, INTmed, and PIE

TE: total effect

CDE: controlled direct effect, the direct effect of the exposure if the mediator were removed

INT_ref_: reference interaction, an additive interaction

INT_med_: mediated interaction, an additive interaction that only operates if the exposure influences the mediator

PIE: pure indirect effect.

**Fig 1:**
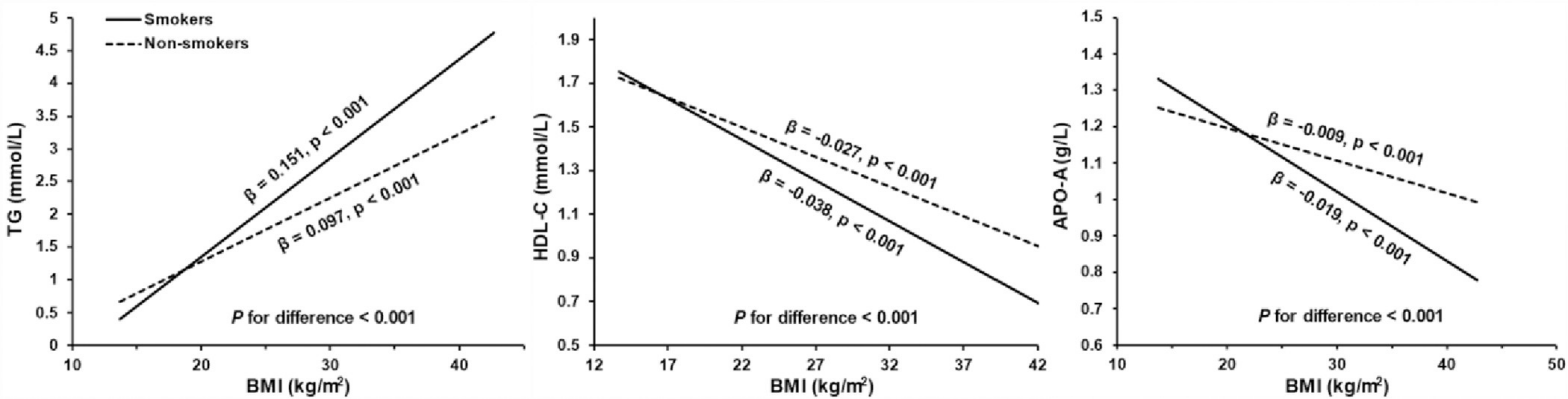
Relationship between body mass index (BMI) and lipid profiles in smokers and non-smokers. TG = triglyceride; HDL-C = high density lipoprotein cholesterol; Apo-A = apolipoprotein A; Covariates for adjustment included age, sex, region, energy intake and education.

**Fig 1** shows the relationships of BMI on lipid profiles in different smoking status, adjusting for age, sex, region, energy intake and education. The positive effect of BMI on TG was higher in smokers than in non-smokers (*β =* 0.151 in smokers versus *β =* 0.097 in non-smokers, *P* for difference < 0.001). In contrast, the negative effects of BMI on HDL-C and APO-A were greater in smokers than in non-smokers (HDL-C: -0.037 versus -0.027, APO-A: -0.019 versus -0.009, *P* for difference < 0.001 for both). The presence of smoking enhanced the effect of BMI on these three lipids.

## Discussion

In this population-based study, we examined the joint effect of smoking and BMI on serum lipid profiles in Chinese adults using the four-way decomposition analysis. We found a significant interaction effect of smoking and BMI on TG, HDL-C and APO-A, but BMI maybe a mediator for the association of smoking with TC, LDL-C and APO-B. These findings provide evidence that the effect of smoking and BMI on serum lipid profiles is complex, and the effect of BMI on lipid profiles is modified by cigarette smoking in Chinese adults.

In the current study, we found smokers had higher TG, APO-B, and lower HDL-C, which was consistent with previous studies [4–6]. We further explored the effect of smoking on lipids in different sex and found that smoking was not associated with any biomarker of interest in females. The low proportion of smokers in females (3.80%) may account for the instability. Recognized mechanisms for the effects of smoking on lipids are summarized as follows: (1) Nicotine in cigarettes alters the release of catecholamine and adrenaline, leading to increased serum levels of free fatty acids in smokers. Free fatty acids could promote liver to secrete extremely low-density lipoprotein and triglycerides. Serum HDL-C levels are negatively correlated with extremely low-density lipoprotein levels [4, 17]. (2) Smoking will induce insulin resistance [18], the activity of lipoprotein lipase (responsible for catalyzing TG hydrolysis and clearing TG) at the skeletal muscle is suppressed by insulin [19]. Finally cause the increase of TG and very low-density lipoprotein (VLDL) [17]. (3) The enzymes such as lecithin cholesterol acyl-transferase and hepatic lipase are directly responsible for maintaining the balance of HDL-C in metabolism, which could promote the accumulation of esterified cholesterol. The activity of these enzymes will be affected by smoking, and the small disruptions will further change the levels of HDL-C in circulation [17, 20, 21]. Together, smoking seriously disturbs the metabolism of lipid profiles, finally increases the risk of dyslipidemia.

The correlation between BMI and lipids are also consistent with published researches [9, 22–24]. Krauss et al. proved a direct correlation between increased BMI and higher TC, LDL-C and TG [25]. The flow of fatty acids to the liver leads to the accumulation of TG. Then the synthesis of VLDL will increase and the lipolysis of chylomicron impeded [26, 27]. Meanwhile, the increased APO-B is associated with the overproduction of lipoproteins [28]. In contrast, many epidemiological studies found a significant inverse correlation between BMI and total HDL-C in adults [22–24]. The enrichment and subsequent hydrolysis of HDL-C may be a potential explanation for lower HDL-C in individuals with high BMI [23, 24]. The relationship between BMI and APO-A is the same as HDL-C because APO-A is a structural protein of HDL-C.

Based on the four-way decomposition, we examined the complicated effect of smoking and BMI on serum lipid profiles. Despite the total effect of smoking on lipid profiles was not all significant, this method was still applicable [29]. There was only indirect effect between smoking and BMI on TC, LDL-C and APO-B, and the corresponding effect size were quite small. The relationship has not been adequately explored in published studies, BMI maybe a potential mediator for the association of smoking with TC, LDL-C and APO-B.

Compared with the pure indirect effect, the interaction effect between smoking and BMI was dominant for TG, HDL-C, and APO-A. In this study, it was found that smoking amplified the role of BMI in reducing HDL-C, APO-A and increasing TG. Research based on the Third National Health and Nutrition Examination Survey also reported a significant interaction effect of smoking and BMI on CVD mortality [11]. A prospective cohort study concluded that obese smokers had a 6 to 11-fold mortality risk of circulatory disease, compared to normal weight, never smokers [30]. Pooled analysis of the Asia Pacific found that smoking modified the positive correlation between BMI and coronary heart disease [31]. The physiological mechanism of the interaction effect between smoking and BMI has not been elucidated, further research is needed in the future. Simultaneous smoking cessation and weight control may have a synthetic effect in reducing the incidence of dyslipidemia. Health authorities should emphasize the importance of both when implementing intervention programs or conducting health education.

The current study has some important strengths. A large representative sample from China was utilized to study the topics of interest. We used the four-way decomposition to determine the effect between smoking and BMI on serum lipid profiles. Stratification analysis verified the interaction effect of smoking and BMI, showing that among smokers, the elevating BMI had a stronger effect on reduced HDL-C, APO-A, and increased TG. At the same time, this research has some limitations. The information related to smoking in CHNS was based on self-reports, and the proportion of smokers in Chinese females was rather small, so the generalization of conclusion was limited. The cross-sectional study is unable to get a clear causal effect. Though the covariates were adjusted, unknown confounding was not considered.

## Conclusions

This study explored the complex effect of smoking and BMI on serum lipid profiles. There was an interaction effect between smoking and BMI on TG, HDL-C, and APO-A, while BMI maybe a mediator for the association of smoking with TC, LDL-C and APO-B. Therefore, in order to reduce the risk of dyslipidemia which could lead to cardiovascular diseases, smoking cessation is necessary, especially for the overweight.

## Supporting information

S1 Table Decomposition of the effect of current smoking on lipid profiles due to mediation and interaction with BMI. (DOCX)

## List of abbreviations

BMI: body mass index
TC: total cholesterol
TG: triglyceride
LDL-C: low density lipoprotein cholesterol
HDL-C: high density lipoprotein cholesterol
Apo-A: apolipoprotein A
Apo-B: apolipoprotein B
CDE: controlled direct effect
INT_ref_: the reference interaction
INT_med_: the mediated interaction
PIE: the pure indirect effect
