# Supplementary material for: Four-way decomposition of effect of cigarette smoking and body mass index on serum lipid profiles
Source: PLoS One. 2022 Aug 18;17(8):e0270486. doi: 10.1371/journal.pone.0270486 (PMC9387787; doi:10.1371/journal.pone.0270486)
Supplement: S1 Table — (DOCX) [file pone.0270486.s001.docx]

**Supporting information**

**Four-way Decomposition of Effect of Cigarette Smoking and Body Mass Index on Serum Lipid Profiles**

Wenhao Yu ^1,2,3^, Chaonan Gao ^1,2,3^, Xiangjuan Zhao ^4^, Chunxia Li ^1,2,3^, Bingbing Fan ^1,2,3^, Jiali Lv ^1,2,3^, Mengke Wei ^1,2,3^, Li He ^1,2,3^, Chang Su ^5^*, Tao Zhang ^1,2,3^*

^1^ Department of Biostatistics, School of Public Health, Cheeloo College of Medicine, Shandong University, Jinan, Shandong, 250012, China

^2^ Institute for Medical Dataology, Shandong University, Jinan, 250002, China

^3^ National Institute of Health Data Science of China, Jinan, 250002, China

^4^ Maternal and Child Health Care of Shandong Province, Cheeloo College of Medicine, Shandong University, Jinan 250012, China

^5^ National Institute for Nutrition and Health, Chinese Center for Disease Control and Prevention, Beijing 102206, China

**Corresponding authors:**

**Tao Zhang,** MD, PhD, Department of Biostatistics, School of Public Health, Cheeloo College of Medicine, Shandong University, PO Box 100, 44 Wenhuaxi Road, Jinan 250012, China. Tel: +86-0531-88382140; E-mail: [taozhang@sdu.edu.cn](mailto:taozhang@sdu.edu.cn)

and **Chang Su**, MD, PhD, National Institute for Nutrition and Health, Chinese Center for Disease Control and Prevention, 155 Changba Road, Beijing 102206, China.

Email: [suchang@ninh.chinacdc.cn](mailto:suchang@ninh.chinacdc.cn) Tel: 0531-88382140

The authors do not have any conflict of interest.

**Supplement Table 1.** Decomposition of the effect of current smoking on lipid profiles due to mediation and interaction with BMI

|  | TE | |  | CDE | |  | INT_ref_ | |  | INT_med_ | |  | PIE | |
| --- | --- | --- | --- | --- | --- | --- | --- | --- | --- | --- | --- | --- | --- | --- |
|  | Est (SE) | *P*-value |  | Est (SE) | *P*-value |  | Est (SE) | *P*-value |  | Est (SE) | *P*-value |  | Est (SE) | *P*-value |
| TC | 0.043 (0.030) | 0.153 |  | -0.281 (0.168) | 0.094 |  | 0.361 (0.167) | 0.031 |  | -0.009 (0.005) | 0.044 |  | -0.028 (0.005) | <0.001 |
| TG | 0.046 (0.020) | 0.018 |  | -0.420 (0.103) | <0.001 |  | 0.513 (0.103) | <0.001 |  | -0.013 (0.003) | <0.001 |  | -0.034 (0.006) | <0.001 |
| LDL-C | -0.001 (0.029) | 0.996 |  | -0.054 (0.165) | 0.742 |  | 0.078 (0.164) | 0.634 |  | -0.002 (0.004) | 0.635 |  | -0.022 (0.004) | <0.001 |
| HDL-C | 0.004 (0.015) | 0.749 |  | 0.229 (0.082) | 0.006 |  | -0.247 (0.082) | 0.003 |  | 0.006 (0.002) | 0.008 |  | 0.017 (0.003) | <0.001 |
| APO-A | 0.034 (0.011) | 0.003 |  | 0.249 (0.064) | <0.001 |  | -0.227 (0.064) | <0.001 |  | 0.006 (0.002) | 0.003 |  | 0.006 (0.001) | <0.001 |
| APO-B | 0.012 (0.008) | 0.137 |  | -0.036 (0.044) | 0.405 |  | 0.061 (0.043) | 0.162 |  | -0.002 (0.001) | 0.175 |  | -0.011 (0.002) | <0.001 |

TC = total cholesterol; TG = triglyceride; LDL-C = low density lipoprotein cholesterol; HDL-C = high density lipoprotein cholesterol; Apo-A = apolipoprotein A; Apo-B = apolipoprotein B;

Est: the effect size of total effect or the component due to CDE, INTref, INTmed, and PIE;

TE: total effect;

CDE: controlled direct effect, the direct effect of the exposure if the mediator were removed;

INT_ref_: reference interaction, an additive interaction;

INT_med_: mediated interaction, an additive interaction that only operates if the exposure influences the mediator;

PIE: pure indirect effect.
